# Prevalence, predictors and pregnancy outcomes of unprescribed and herbal medicine use in Ibadan, Nigeria

**DOI:** 10.1186/s12906-023-03838-8

**Published:** 2023-01-20

**Authors:** Ikeola Adeoye, Victoria Etuk

**Affiliations:** 1 Department of Epidemiology and Medical Statistics, Faculty of Public Health, College of Medicine, University of Ibadan, Ibadan, Nigeria; 2 Consortium of Advanced Research for Africa (CARTA), Nairobi, Kenya

**Keywords:** Unprescribed medicines, Herbal medicines, Prevalence, Predictors, Pregnancy outcomes, Nigeria

## Abstract

**Background:**

Unprescribed and herbal medicines use among pregnant women is a public health concern in low and middle-income countries because of the potential teratogenic effects, insufficient safety and weak drug regulatory systems. Unprescribed and herbal medicines are common among pregnant women in Nigeria, and only a few researchers have documented the prevalence and the risk factors. However, evidence on the associated pregnancy outcomes is still lacking. We assessed the prevalence, predictors and pregnancy outcomes of unprescribed and herbal medicines use among pregnant women in Ibadan, Nigeria.

**Methods:**

This study was a component of the Ibadan Pregnancy Cohort Study, a prospective cohort study, among pregnant women in Ibadan, Nigeria, who were enrolled at ≤ 20 weeks gestation at their first antenatal visit and followed up till delivery. In all, 571 women participated in the maternal drug use assessment during the third trimester using a pretested interviewer-administered questionnaire. The primary outcomes were unprescribed and herbal medicines use and pregnancy outcomes, secondary outcomes, were abstracted from medical records. The predictors assessed included sociodemographic, obstetric, antenatal care utilization, and lifestyle characteristics. Bi-variate logistic and Poisson regression analyses were used to evaluate the predictors and relative risk for the pregnancy outcomes of unprescribed and herbal medicines at 5% significance.

**Results:**

The prevalence of unprescribed and herbal medicine use was 31.9% and 21.7%, respectively. On bivariate analysis, the significant predictors of unprescribed medicine (which were protective) were tertiary education, increasing income, adequate antenatal care (≥ 4 visits), and at least two doses of sulfadoxine-pyrimethamine. However, high parity and having an antenatal admission increased the risk. However, after adjusting for confounders, the significant factors associated with unprescribed medicines were; tertiary education (AOR) = 0.23; 95% CI: (0.06 – 0.95); p-value: 0.043] and obtaining at least two doses of sulfadoxine-pyrimethamine [AOR = 0.33; 95% CI: (0.29 – 3.60); p-value: 0.048]. For herbal medicines, the predictors were similar to unprescribed drugs. However, after adjusting for confounders, none was significant for herbal medicines. Unprescribed and herbal medicines were not significantly associated with pregnancy outcomes.

**Conclusions:**

Unprescribed and herbal medicines use were common among pregnant women in Ibadan, Nigeria, particularly among women with low economic status and those with poor utilization of antenatal care services. These significant predictors can be targeted for public health intervention. Specifically, health education that discourages the use of unprescribed and herbal medications to pregnant women during antenatal care.

## Introduction

Medication use in pregnancy is a public health concern because of the potential adverse consequences on the mother and developing foetus, especially the possible teratogenic effects during organogenesis [1, 2]. Even after organogenesis, when the possibility of congenital abnormalities is much less, it may affect the maturation of foetal organs [1, 3]. Also, since pregnant women are often excluded from clinical trials of medicines (whether prescription drugs, over-the-counter or herbal medicine) for ethical reasons, therefore there is insufficient evidence on the safety and benefit of many medications in pregnancy [4, 5]. Hence the associated adverse effects of drug use during pregnancy have often been discovered after delivery or during post-marketing surveillance because of insufficient safety data for both mother and foetus [6, 7]. Pregnant women usually require pharmacological treatment for acute, pre-existing chronic or pregnancy-related medical conditions [5, 6, 8, 9]. Medication use among pregnant women has increased globally [1, 10], For example, a web-based study across four continents reported 81.2% of at least one medication during pregnancy [8]. Unprescribed medicines are described as the use of pharmaceutical products without medical prescription to treat symptoms or self-diagnosed health issues [11]. In contrast, herbal medicines are plant-derived materials or preparations with therapeutic benefits and contain raw or processed ingredients from one or more plants [11]. Herbal medicines include herbs, herbal materials, herbal preparations and finished herbal products that contain active ingredients, parts of plants, other plant materials, or combinations [12].

Unprescribed medicine (UM) and herbal medicine use is a public health challenge reported among pregnant women in high-income and low-income countries. In high-income countries such as the USA, Canada and Australia, the prevalence of unprescribed medicine use in pregnancy was between 39.2% and 81.8% [8]. In low-and middle-income countries (Indonesia, Tanzania, Ethiopia, Uganda and Ghana), the estimated prevalence was between 11%—43.9%. [13–17]. Researchers in Nigeria have also reported a high prevalence of unprescribed medicine use, ranging from 62.9% to 72.4% across different states [18–21]. Similarly, the prevalence of herbal medicine (HM) use is also high among pregnant women. A multinational study involving 23 countries from Europe, North and South America and Australia reported an average prevalence of 28.9% HM use among pregnant women, with the highest and lowest rates found in Russia (68.9%) and Sweden (4.3%), respectively [22]. Other studies have reported herbal medicine use among pregnant women in the Netherlands (9.4%) [13], and Italy (36.4%) [14]. Higher prevalences of maternal herbal medicine use have been reported in countries in the African continent, with estimates ranging between 36.8% in Nigeria, and 73% in Ethiopia [15]. Common unprescribed medicines used by pregnant women include analgesics, antibiotics and antimalarial among pregnant women [3, 4, 16, 17]. For herbal medicines, Ginger *(Zingiber officinale)*, cranberry, and garlic *(Allium sativum)* are some of the most familiar herbal medicines in high-income countries worldwide [18, 19]. Similarly, Duru et al. (2016) reported bitter leaf, bitter kola, neem leaf, garlic, ginger, and scent leaf as the frequently used HM among Nigerian pregnant women [20]. The factors associated with UM and HM use in pregnancy include age, parity, education, occupation, income, marital status, rural residence, history of maternal illness and unplanned pregnancy [4, 18, 20, 21, 23–25].

Inappropriate drug use, such as unprescribed and herbal medicines, widespread among pregnant women can increase the risk of adverse pregnancy outcomes [6]. There is increasing evidence that maternal use of non-prescribed and herbal medicines can cause significant harm to both the mother and foetus. This could lead to adverse maternal and foetal outcomes such as preterm birth, low birth weight, congenital malformations and attention-deficit disorder [16, 26, 27]. Similarly, Balbontin et al. (2019) in a systematic review of maternal HM, reported the association between herbal medicine use and adverse perinatal outcomes and demonstrated a higher risk of preterm births, caesarean section and maternal morbidity [28]. This is disturbing, particularly in a country like Nigeria, with a maternal mortality ratio (MMR) of 580 maternal deaths per 100,000 live births, child mortality of 128 deaths per 1000 live births and neonatal mortality rate of 35 deaths per 1000 live births [29]. Nigeria's maternal care services align with the WHO guidelines, stipulating that at least four antenatal care visits are needed to provide preventive measures, interventions and quality care necessary for a healthy pregnancy experience [30, 31].

Furthermore, there is weak enforcement of regulations on the sale and distribution of herbal and prescription medicines in Nigeria [32]. These regulatory challenges have led to the spread of drugs, easy access to medication from undocumented sources, and a lack of enforcement of standards on the sale and distribution of herbal medicines [33]. Weak regulations also imply that women who use unprescribed medicines may be exposed to the consumption of counterfeit drugs and toxic herbal preparations [34, 35], thus posing a risk to their fetuses. Unregulated use of unprescribed medicines such as antibiotics may lead to antimicrobial resistance and pose a risk during pregnancy [36]. Hence, UM and HM are essential contributors to adverse perinatal outcomes, maternal and child morbidity and mortality. Even though UM and HM are modifiable factors, they have not been given sufficient attention in Nigeria's maternal and child health services. Although the prevalence and risk factors of UM and HM use have been investigated in Nigeria, the associated pregnancy outcomes have received scant attention. Understanding the effects of non-prescribed medicine use on the pregnant population in Nigeria can help generate evidence for the safety of medicines in Nigeria. This can help guide policy interventions in maternal and child care. Therefore we investigated the prevalence, predictors and associated pregnancy outcomes of UM and HM use among pregnant women in Ibadan, Nigeria.

## Materials and methods

### Study design and sampling technique

This study is a component of the Ibadan Pregnancy Cohort Study (IbPCS), a prospective cohort study conducted among pregnant women accessing maternal care in Ibadan, the capital city of Oyo State and Nigeria. This study spanned from April 2018 to September 2019. IbPCS investigated maternal obesity and lifestyle factors' associations (including maternal drug use) with cardio-metabolic and pregnancy outcomes among women and their offspring. The methodology details (study population, sample size and selection) have been reported elsewhere [37]. In brief, we recruited women in early gestation (≤ 20 weeks) during their antenatal booking visit from four comprehensive obstetric facilities within the Ibadan metropolis; University College Hospital, Adeoyo Maternity Teaching Hospital, Jericho Specialist Hospital and Saint Mary Catholic Hospital, Ibadan. These facilities are the main referral centres for complex obstetric cases within the Ibadan metropolis. In all, one thousand seven hundred and forth-five women were recruited at baseline who met the eligibility criteria, including women ≤ 20 weeks' gestation, aged ≥ 18 years, and women without severe medical complications. These women were followed up from booking them up to delivery. Data were collected using pretested, interviewer-administered questionnaires and desktop review of medical records at three points during the study – booking, third trimester, and delivery. We examined maternal drug use and health care utilisation in the third trimester, and pregnancy outcomes were ascertained at delivery from delivery records. Only 571 women participated in the maternal drug use assessment during the third trimester out of the 1,745 women enrolled at their first antenatal booking visits. The estimated power calculations using the following parameters: prevalence of unprescribed medicines (31.9%) which is the primary outcome, type I error of 5% (Z(α/2) = 1.96), resulting in a power = 0.9095. We obtained ethical approval for this study from the University of Ibadan/University College Hospital (UI/UCH), Institutional Review Board (UI/EC/15/0060) and Oyo State Ministry of Health Ethical Committee (AD/13/479/710). Verbal and written informed consent was obtained from respondents before recruitment into the study. The study protocol and conduct adhered to the principles in the Declaration of Helsinki.

### Measures

The primary outcome variables were unprescribed medicines and herbal medicine use. We assessed drug use in the index pregnancy in the third trimester. Unprescribed medicines refer to any orthodox medicine not prescribed by the physician or health worker but taken on the woman's own accord. Unprescribed medications were obtained by self-report [17, 21, 38] in response to specific questions reported below 1.) Have you taken any unprescribed medicines during this pregnancy 2.) If yes, which unprescribed medicines did you take? This was to assess the type of drugs taken. 3.) If yes, why did you take the drug of the unprescribed medicine? Which was to evaluate the reason for which the drug was taken. Herbal medicine use was also obtained by self-report [18, 20, 39] in response to specific questions reported below 1.) Have you taken any herbal preparation during this pregnancy 2.) If yes, which herbal preparation did you take; this was to assess the type of herbal drugs taken. 3.) If yes: why did you take the herbal preparation; this was to evaluate why the herbal drug was taken. In addition, information on routine antenatal medicines was also assessed: haematinics, tetanus toxoid, folic acid intake, and Intermittent Presumptive Treatment with Sulfadoxine-Pyrimethamine (IPT-SP).

### Exposure variables

The explanatory variables examined [23, 38, 40] were maternal age (< 35 years, ≥ 35 years), marital status (single or married), level of education (primary, secondary or tertiary), employment status (employed, unemployed), religion (Christianity, Islam), average monthly income level in Naira (< 20,000, 20,000 – 99,999; ≥ 100,000). Lifestyle characteristics such as alcohol consumption (yes or no), exposure to tobacco (yes or no), pre-pregnancy contraceptive use (yes or no), pregnancy-related characteristics including parity (0, 1–3, ≥ 4), gravidity (1, 1–4, ≥ 5), number of antenatal care visits (< 4, ≥ 4), folic acid use (yes or no), doses of tetanus toxoid (< 2, ≥ 2), and IPT-SP received (< 2, ≥ 2). Furthermore, others were a history of chronic medical illness (yes or no) and hospital admission in the current pregnancy (yes or no).

### Pregnancy outcomes

Pregnancy Outcomes: i.) Caesarean section: refers to the mode of delivery of the infant achieved through an abdominal surgical procedure which could be elective or emergency; ii.) Spontaneous vertex delivery: delivery of the child per vaginum; iii.) Macrosomia: refers to infants with birthweight ≥ 4.0 kg, iv.) Low birth weight (LBW) was defined as < 2.5 kg. vi.) Preterm birth occurred before 37 completed weeks of gestation vii.) Birth Asphyxia: defined as infants with APGAR score ≤ 7 at one minute; viii.) Postpartum Haemorrhage: Blood loss ≥ 500 MLS post-vaginal delivery and ≥ 1000mls post-caesarean section; vi.) Raised blood pressure (BP): described as systolic BP ≥ 140 mmHg or/and diastolic BP ≥ 90 mmHg. Pregnancy outcomes [41, 42] were ascertained during delivery from medical records evaluated at the end of the pregnancy.

### Statistical analysis

Statistical analysis was performed using STATA version 13. Summary statistics were used, and proportions were reported for categorical variables. Frequency distribution of the study participants and prevalence proportions of UM and HM by sociodemographic, obstetric and lifestyle characteristics were also reported. The classification of unprescribed medicines according to the WHO ATC classification was displayed using a bar graph. We conducted bivariate logistic regression analyses to test the associations between the outcome and the explanatory variables. The unadjusted and adjusted odds ratios, 95% confidence intervals and p-values were reported. Factors included in the final model were those found significant (*p* < 0.05) at the bivariate level. For the multivariate analysis, the dependent variables were unprescribed medicines and herbal preparation use. The incidence of pregnancy outcomes was obtained and examined for significant association using Poisson regression with relative risk (RR) and 95% confidence intervals. The pregnancy outcomes looked at variables were: caesarean section, spontaneous vertex delivery, induction of labour, low birth weight, macrosomia, birth asphyxia, preterm delivery and postpartum haemorrhage.

## Results

### Characteristics of pregnant women by unprescribed and herbal medicines

**Fig. 1:**
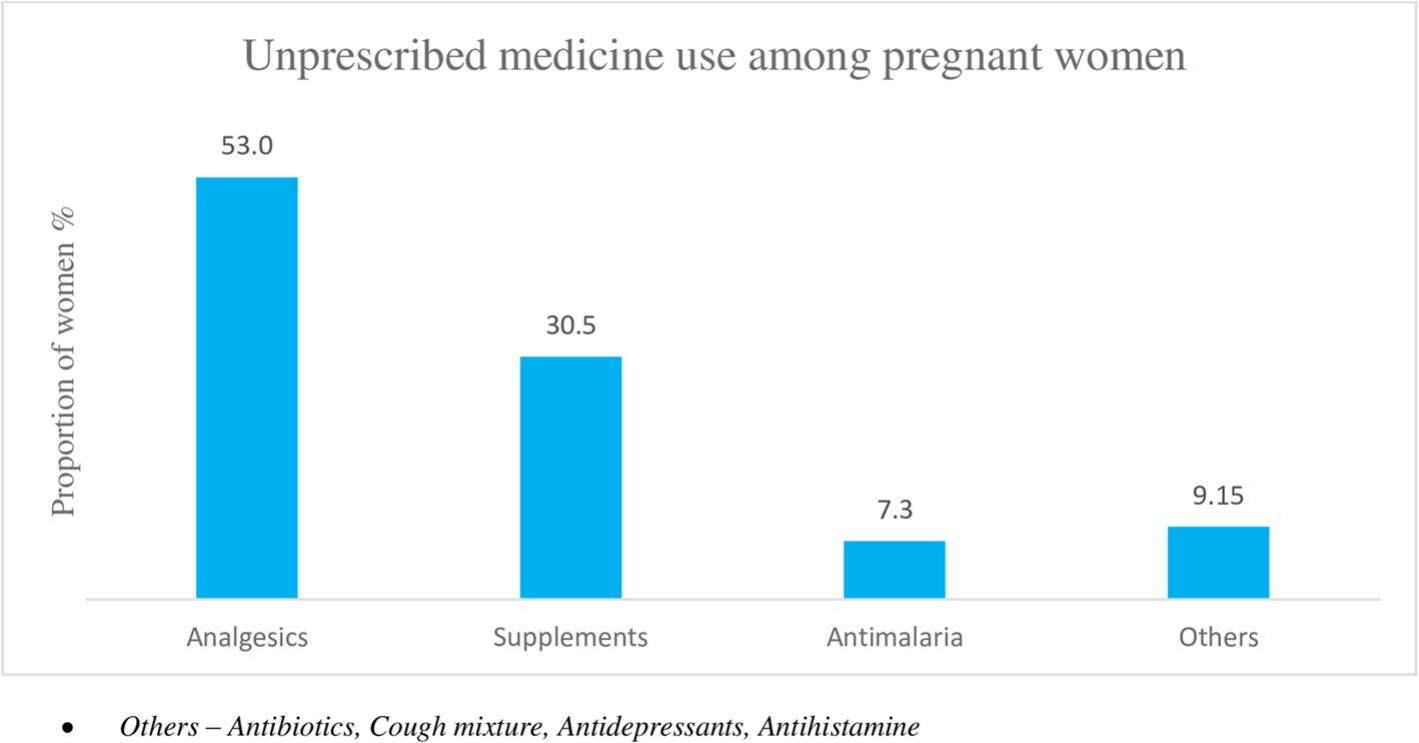
Pattern of unprescribed drug use by the pregnant women

**Fig. 2:**
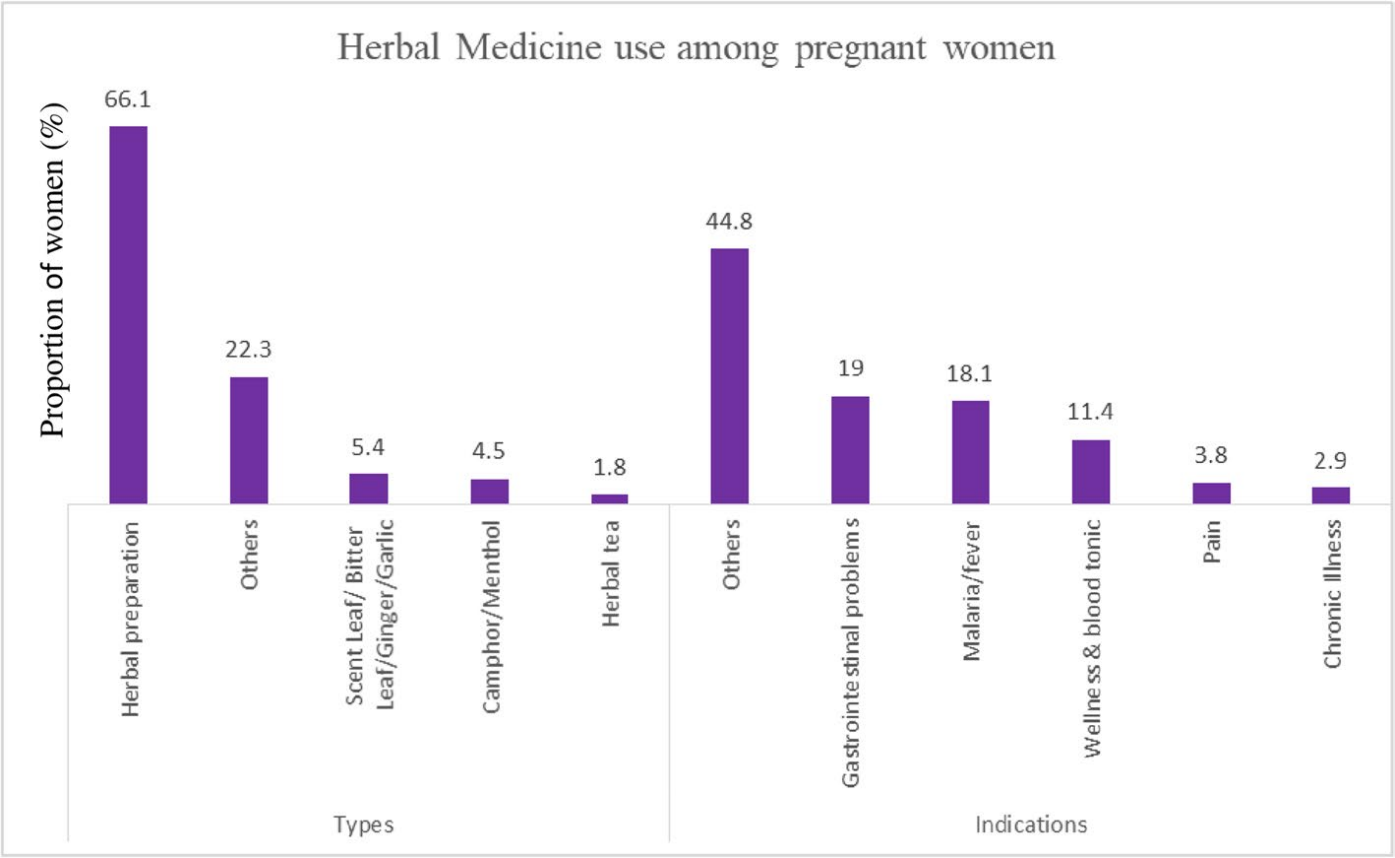
Pattern and indications of herbal medicine use by the pregnant women

The characteristics of pregnant women by unprescribed and herbal medicines use and the prevalence is described in Table 1. In the third trimester, five hundred seventy-one pregnant women participated in the maternal drug use assessment. The prevalences of unprescribed and herbal medicine use were 31.9% and 21.7%, respectively. Unprescribed medicines were less common among women with at least 4 ANC visits (27.2%), at least two doses of TT vaccination (27.3%), at least two doses of IPT-SP-SP (21.2%), folic acid supplements (2.3%) and women who were hospitalised during antenatal period (23.8%). In contrast, the prevalence was higher among women with parity ≥ 4 (57.1%), alcohol use during pregnancy (38.2%), tobacco exposure (42.9%), and women who practised Islam (40.6%). The prevalence of unprescribed medicines declined with education [primary (66.7%), secondary (61.4%), and tertiary (25.8%)] and income [< 20,000 (45.2%), '20,000 – 99,999" (29.6%), ≥ 100,000 (21.4%)]. Similarly, herbal medicines were also more frequent among women with less than 4 ANC visits (35.7%), less than two doses of TT vaccination (22.2%), and less than two doses of IPT-SP: (25.9%). The commonly used unprescribed medicines were analgesics (53.5%), multivitamins supplements (30.5%) and anti-malaria medications (Fig. 1). Herbal preparations (66.1%) were the most familiar type of HM, while gastrointestinal problems (19.0%) and malaria (18.1%) were the commonest indications for their use (Fig. 2).

**Table 1:** Characteristics of pregnant women by unprescribed and herbal medicines use

		Unprescribed medicines	Herbal medicines
	Total *(N)*^ab^	*Prevalence (%)*	*Prevalence (%)*
**Overall**	571	182 **(31.9%)**	112 **(21.7%)**
**Age**			
Less than 35 years	454 (79.5)	145 (31.9)	93 (22.3)
35 and above	117 (20.5)	37 (31.6)	19 (18.5)
**Education**			
Primary	9 (1.6)	6 (66.7)	2(40.0)
Secondary	88 (15.4)	54 (61.4)	37 (49.3)
Tertiary	473 (83.0)	122 (25.8)	73 (16.6)
**Employment status**			
Not employed	54 (9.5)	13 (24.1)	12(22.2)
Employed	517 (90.5)	169 (32.7)	100 (21.4)
**Religion**			
Christianity	410 (71.9)	117 (28.5)	54 (15.0)
Islam	160 (28.1)	65 (40.6)	56 (35.2)
**Marital Status**			
Single	28 (4.9)	10 (35.7)	10 (35.7)
Ever Married	543 (95.1)	172 (31.7)	102 (20.7)
**Monthly Income (Naira)**			
Less Than 20,000	115 (22.7)	52 (45.2)	41 (38.3)
20,000–99,999	335 (66.2)	99 (29.6)	49 (16.3)
100,000 And Above	56 (11.1)	12 (21.4)	7 (14.0)
***Pregnancy related Factors***			
**Parity**			
Nulliparous	264 (46.5)	78 (29.6)	50 (20.5)
1–3	283 (49.8)	92 (32.5)	58 (22.2)
4 And Above	21 (3.7)	12 (57.1)	4 (30.8)
**Number of ANC visits**			
< 4	83 (23.4)	41 (49.4)	25 (35.7)
≥ 4	272 (76.6)	74 (27.2)	48 (19.1)
**Doses of tetanus toxoid**			
< 2	60 (12.2)	23 (38.3)	12 (22.2)
≥ 2	433 (87.8)	118 (27.3)	75 (18.6)
**Doses of IPT-SP**			
< 2	87 (20.0)	32 (36.8)	21 (25.9)
≥ 2	349 (80.0)	74 (21.2)	46 (13.8)
**Routine haematinics**			
No	43 (8.2)	3 (7.0)	4 (9.1)
Yes	481 (91.8)	151 (31.4)	93 (20.1)
**Folic acid supplements**			
No	87 (16.5)	2 (2.3)	0 (0.0)
Yes	440 (83.5)	145 (32.9)	76 (19.6)
**Antenatal hospital admission**			
No	330 (75.9)	21 (6.4)	7 (2.1)
Yes	105 (24.1)	25 (23.8)	11 (11.3)
**Chronic medical illness**			
No	500 (87.6)	164 (32.8)	12 (17.4)
Yes	71 (12.4)	18 (25.5)	100 (22.1)
***Lifestyle Factors***			
**Alcohol consumption**			
No	495 (86.7)	153 (30.9)	100 (21.9)
Yes	76 (13.3)	29 (38.2)	12 (18.8)
**Tobacco exposure**			
No	550 (96.3)	173 (31.5)	109 (21.6)
Yes	21 (3.7)	9 (42.9)	3 (17.6)

^a^Frequency distribution of study participants

^b^Total for HM users 521

### Factors associated with maternal unprescribed and herbal medicines use

Table 2 shows the predictors of unprescribed medicine among pregnant women in Ibadan, Nigeria. Women who had tertiary education [crude odds ratio (OR) = 0.17; 95% CI: (0.04–0.71); *p*-value: 0.014] had lower odds for UM use than women with primary education. Similarly, the increasing income also lower the odds of UM use '20,000 – 99,999' [(OR) = 0.51; 95% CI: (0.33–0.79); *p*-value: 0.002] and ≥ 100,000 [(OR) = 0.33; 95% CI: (0.16–0.69); *p*-value: 0.003], than women who earned < 20,000. Also, women with ≥ 4 ANC visits [(OR) = 0.38; 95% CI: (0.23–0.64); *p*-value: < 0.0001] were less likely to use UM than women with < 4 visits. Grand multiparous women [OR = 3.18; 95% CI: (1.29–7.85); *p*-value: 0.012], being a Muslim [(OR) = 1.71; 95% CI: (1.17–2.50); *p*-value: 0.006] and having a history of antenatal hospital admissions [(UOR) = 4.60; 95% CI: (2.45–8.63); *p*-value: < 0.001] were associated with a higher likelihood of UM use. However, on multivariate logistic regression, women with tertiary education [adjusted odds ratio (AOR) = 0.23; 95% CI: (0.06 – 0.95); *p*-value: 0.043] and having at least two doses of IPT-SP [AOR) = 0.33; 95% CI: (0.10 – 0.99); *p*-value: 0.048] were associated with UM use after adjusting for confounders.

**Table 2:** Predictors of unprescribed medicines use among pregnant women in Ibadan, Nigeria

	**Crude OR (95% CI)**	***p*-value**	**Adjusted OR (95% CI)**	***p*-value**
**Characteristics**				
**Age**				
Less than 35	1			
35 and above	0.99 (0.64—1.53)	0.948		
**Education**				
Primary or less	1		**1**	
Secondary	0.79 (0.19 – 3.39)	0.755	0.59 (0.19 – 3.29)	0.655
Tertiary or higher	0.17 (0.04—0.71)	**0.014**	0.23 (0.06 – 0.95)	**0.043**
**Employment status**				
Not employed	1			
Employed	1.53 (0.80 – 2.93)	0.199		
**Religion**				
Christianity	1		1	
Islam	1.71 (1.17—2.50)	**0.006**	0.91 (0.27 – 3.10)	0.266
**Marital status**				
Single	1			
Ever married	0.83 (0.38—1.84)	0.655		
**Income (monthly income in naira)**				
Less than 20,000	1		1	
20,000–99,999	0.51 (0.33—0.79)	**0.002**	0.39 (0.12 – 1.27)	0.119
100,000 and above	0.33 (0.16 – 0.69)	**0.003**	0.17 (0.01 – 1.60)	0.121
**Parity**				
Nulliparous	1		1	
1–3	1.15 (0.80—1.65)	0.454	1.14 (0.40 – 3.23)	0.812
4 and above	3.18 (1.29 – 7.85)	**0.012**	2.14 (0.19 – 3.29)	0.255
**Number of ANC visits**				
< 4	1		1	
≥ 4	0.38 (0.23 – 0.64)	** < 0.001**	1.02 (0.29 – 3.60)	0.973
**Doses of tetanus toxoid**				
< 2	1			
≥ 2	0.60 (0.34—1.05)	0.077		
**Doses of IPT-SP**				
< 2	1		1	
≥ 2	0.46 (0.28 – 0.77)	**0.003**	0.33 (0.10 – 0.99)	**0.048**
**Antenatal admission**				
No	1		1	
Yes	4.60 (2.45 – 8.63)	** < 0.001**	2.16 (0.69 – 6.76)	0.185
**Chronic medical illness**				
No	1			
Yes	0.69 (0.39 – 1.23)	0.209		
***Lifestyle Factors***				
**Alcohol consumption**				
No	1			
Yes	1.38 (0.83 – 2.27)	0.208		
**Tobacco exposure**				
No	1			
Yes	1.63 (0.68 – 3.95)	0.275		

The predictors of herbal medicine use among pregnant women in Ibadan, Nigeria, are shown in Table 3. Muslims, compared to Christians, had high odds for UM use [(OR) = 3.07; 95% CI: (1.99–4.75); *p*-value: 0.001]. In contrast, increasing income was also associated with less likelihood for HM use: '20,000 – 99,999' [(OR) = 0.31; 95% CI: (0.091–0.52); *p*-value: < 0.001] and ≥ 100,000 [(OR) = 0.26; 95% CI: (0.11–0.64); *p*-value: 0.003], than women who earned < 20,000. Women who had ≥ 4 ANC visits [(OR) = 0.43; 95% CI: (0.24–0.76); p-value: 0.004] and ≥ 2 doses of IPT-SP [(OR) = 0.45; 95% CI: (0.25–0.82); *p*-value: 0.009] were less likely to use herbal medicines. On multivariate analysis, none of the factors remained associated with HM use.

**Table 3:** Predictors of herbal medicine use among pregnant women in Ibadan, Nigeria

	**Crude OR (95% CI)**	***p*-value**	**Adjusted OR (95% CI)**	***p*-value**
**Characteristics**				
**Age**				
Less than 35	1			
35 and above	0.79 (0.46—1.37)	0.401		
**Education**				
Primary or less	1			
Secondary	1.46 (0.23 – 9.25)	0.687		
Tertiary or higher	0.30 (0.05 – 1.82)	0.190		
**Employment status**				
Not employed	1			
Employed	0.95 (0.48 – 1.88)	0.891		
**Religion**				
Christianity	1		1	
Islam	3.07 (1.99 – 4.75)	0.001	2.23 (0.58 – 8.52)	0.242
**Marital status**				
Single	1			
Ever married	0.47 (0.21 – 1.05)	0.065		
**Income (monthly income in naira)**				
Less than 20,000	1		1	
20,000–99,999	0.31 (0.91 – 0.52)	** < 0.001**	0.49 (0.11 – 2.24)	0.360
100,000 and above	0.26 (0.11 – 0.64)	**0.003**	0.38 (0.04 – 4.13)	0.430
**Parity**				
Nulliparous	1			
1–3	1.11 (0.72—1.70)	0.636		
4 and above	1.72 (0.51 – 5.83)	0.381		
**Number of ANC visits**				
< 4	1			
≥ 4	0.43 (0.24 – 0.76)	**0.004**	1.20 (0.22 – 6.56)	0.833
**Doses of tetanus toxoid**				
< 2	1			
≥ 2	0.80 (0.40—1.59)	0.526		
**Doses of IPT-SP**				
< 2	1		1	
≥ 2	0.45 (0.25 – 0.82)	**0.009**	0.55 (0.13 – 2.34)	0.415
**Antenatal admission**				
Yes	1		1	
No	5.90 (2.22 – 15.68)	0.000	2.72 (0.66 – 11.27)	0.167
**Chronic medical illness**				
No	1			
Yes	0.74 (0.38 – 1.44)	0.374		
***Lifestyle Factors***				
**Alcohol consumption**				
No	1			
Yes	0.82 (0.42 – 1.60)	0.568		
**Tobacco exposure**				
No	1			
Yes	0.77 (0.22—2.75)	0.695		

### Pregnancy outcomes associated with maternal unprescribed and herbal medicines use

The models showing the incidence proportions and association between unprescribed medicine use and pregnancy outcomes are shown in Table 4. The incidence of the caesarian section was lower among UM users than non-users (25.6% vs 42.0%), and a 39% lower risk in UM users [Relative risk (RR) = 0.61; 95% CI: (0.46–0.80); p-value: 0.001]. The incidence of SVD was significantly higher among UM users compared with non-users [RR = 1.30; 95% CI: (1.11–1.52); *p*-value: 0.001]. There was no association between UM use and macrosomia, induction of labour, low birth weight, postpartum haemorrhage, birth asphyxia and preterm delivery. After controlling for confounding variables, we found no significant association between unprescribed medicine use and pregnancy outcomes. Table 5 presents the association between HM use and pregnancy outcomes. The incidence of preterm delivery (18.3% versus 12.9%), birth asphyxia (22.7% versus 15.4%) and postpartum haemorrhage (21.8% versus 14.9%) were higher among women who used herbal medicines compared with non-users. However, the incidence of GDM (18.6% versus 22.9%) was slightly lower among herb users than non-users. However, these relationships were not statistically significant.

**Table 4:** Association between unprescribed medicines and pregnancy outcomes in Ibadan, Nigeria

**Pregnancy Outcomes**	**Incidence** **(%)**	**Unadjusted** **Relative Risk 95% CI**	***P*-value**	**Adjusted** **Relative Risk 95% CI**	***P*-value**
**Caesarean section**^**a**^					
*Use of UM*	25.6	0.61 (0.46 – 0.80)	**0.001**	0.33 (0.10 – 1.05)	0.061
*Non-use of UM*	42.0	1.00		1.00	
**Spontaneous Vertex Delivery**^**b**^					
*Use of UM*	67.5	1.30 (1.11– 1.52)	**0.001**	1.23 (0.65– 2.34)	0.528
*Non-use of UM*	51.9	1.00		1.00	
**Induction of Labour**					
*Use of UM*	4.5	0.96 (0.40 – 2.29)	0.929		
*Non-use of UM*	4.6	1.00			
**Macrosomia**					
*Use of UM*	7.5	1.31 (0.64 – 2.66)	0.460		
*Non-use of UM*	5.8	1.00			
**Low birth weight**					
*Use of UM*	4.1	0.45 (0.20 – 1.01)	0.052		
*Non-use of UM*	9.2	1.00			
**Preterm delivery**					
*Use of UM*	11.3	0.92 (0.53 – 1.57)	0.748		
*Non-use of UM*	12.3	1.00			
**Birth Asphyxia**					
*Use of UM*	21.4	1.41 (0.89 – 2.23)	0.147		
*Non-use of UM*	15.2	1.00			
**GDM**					
*Use of UM*	22.4	0.98 (0.60 – 1.58)	0.926		
*Non-use of UM*	23.0	1.00			
**Postpartum Haemorrhage**					
*Use of UM*	12.7	0.81 (0.51 – 1.31)	0.393		
*Non-use of UM*	15.7	1.00			

^a^Caesarean section – adjusted for maternal education, religion, income, parity, antenatal visits, antenatal hospital admission

^**b**^Spontaneous vertex delivery – adjusted for maternal education, religion, income, parity, antenatal visits, antenatal hospital admission

**Table 5:** Association between herbal medicines and pregnancy outcomes in Ibadan, Nigeria

**Pregnancy Outcomes**	**Incidence (%)**	**Unadjusted** **Relative Risk 95% CI**	***P*-value**	**Adjusted** **Relative Risk 95% CI**	***P*-value**
**Caesarean section**					
*Use of HM*	32.0	0.81 (0.60 – 1.09)	0.159		
*Non-use of HM*	39.7	1.00			
**Spontaneous vertex delivery**					
*Use of HM*	65.3	1.23 (0.92– 1.62)	0.154		
*Non-use of HM*	53.3	1.00			
**Induction of labour**					
*Use of HM*	2.0	0.38 (0.10 – 1.49)	0.163		
*Non-use of HM*	5.2	1.00			
**Macrosomia**					
*Use of HM*	6.7	1.17 (0.49 – 2.82)	0.724		
*Non-use of HM*	5.8	1.00			
**Low birth weight**					
*Use of HM*	5.6	0.62 (0.25 – 1.52)	0.296		
*Non-use of HM*	9.0	1.00			
**Preterm delivery**					
*Use of HM*	18.3	1.42 (0.85 – 2.37)	0.184		
*Non-use of HM*	12.9	1.00			
**Birth Asphyxia**					
*Use of HM*	22.7	1.47 (0.88 – 2.45)	0.140		
*Non-use of HM*	15.4	1.00			
**Gestational Diabetes Mellitus**					
*Use of HM*	18.6	0.82 (0.44 – 1.50)	0.510		
*Non-use of HM*	22.9	1.00			
**Postpartum Haemorrhage**					
*Use of HM*	21.8	1.46 (0.93 – 2.29)	0.10		
*Non-use of HM*	14.9	1.00			

## Discussion

This paper describes the prevalence and predictors of maternal UM and HM and the association with pregnancy outcomes in Ibadan, southwest Nigeria. Our study's prevalence of UM was 31.9%, indicating that one out of every three women used UM during pregnancy. The prevalence of UM in our study population is similar to those reported in other African countries [21, 40] but higher than the prevalence in Indonesia [23] and Delta State, Nigeria[43]. However, higher prevalences have been reported in Tanzania [44] and other parts of Nigeria [41, 45–47]. Moreover, Bello et al. (2011), in a study investigating maternal drug use among antenatal care attendees in a similar setting as ours about a decade ago, reported a prevalence of 19.2%, which might suggest a possible rise in the prevalence of UM [48]. These variations in prevalence may result from methodological differences, study population and setting, varying drug regulatory mechanisms in different countries, socio-cultural differences and tradition. We found that the prevalence of UM was higher among women with lower socioeconomic status, higher parity (≥ 4), and women who consumed alcohol or had exposure to tobacco during pregnancy. Additionally, the prevalence of UM in this study may have been underestimated due to the under-reporting of UM attributable to social desirability bias and recall bias. Besides, the use of UM is usually higher in the first trimester compared with the third when we assessed maternal drug use in our study [3]. In our research, the most commonly consumed UM were analgesics (paracetamol), supplements (multivitamins) and antimalarial. These medications are not high risk medications during pregnancy, as many of them are considered safe during pregnancy. Paracetamol is reportedly the first-line analgesic and antipyretic among pregnant women [16, 48], although, other studies have reported antibiotics and antimalarials as being more commonly used among pregnant women [44, 49].

In the bivariate analysis, the factors associated with UM use were education, income, religion, parity, IPT-SP use and antenatal hospital admission. Socioeconomic status measured by education and income had an inverse association with UM, indicating that women's access to education and economic opportunities could increase their knowledge about and purchasing power of appropriate medicines. Specifically, women with tertiary education and higher income (≥ 100,000) were 83% and 67% less likely to use UM, respectively. Women with higher-level education are more likely to possess higher health literacy and greater knowledge of the adverse effects associated with unprescribed medicines [23, 50]. The inverse relationship between education and UM has been reported by several studies [17, 23, 41, 44, 48]. For example, Navaro et al. (2018) found that women with a higher SES were more likely to have better knowledge of UM's potential hazard and more drug-related information, including appropriate drug use during pregnancy [9] Hence, providing correct knowledge on drug use and the associated adverse effects of UM on women and their unborn children through educational programs can positively influence pregnant women on safe drug use [51]. Also, higher education affords higher-paying jobs; these women can pay for their health care needs "out-of-pocket" (the most typical means of paying for healthcare in Nigeria) [52]. Therefore pregnant women who earn less may not be able to afford healthcare services and may resort to self-medication.

We also found that women who had at least four antenatal care visits (OR = 0.38) and took at least two doses of intermittent preventive treatment of malaria with IPT-SP (OR = 0.46) were less likely to use UM. Indicating sufficient ANC contacts and uptake of ANC services such as malaria prevention could hamper UM use during pregnancy. Antenatal care is one of the critical interventions for improving maternal and neonatal outcomes, especially in developing countries. It also provides the platform for health-promoting services through education, nutritional support and cessation of substance abuse [53]. The WHO stipulates that at least four visits where essential services are provided is required for a healthy pregnancy experience [30]. Hence, ANC should be targeted for informing pregnant women on medication use, including unprescribed medicines during pregnancy and other substances like alcohol and tobacco, which had higher odds among women who used UM. Dakota et al. (2017) demonstrated, among pregnant women in Nepal, that providing correct knowledge on drug use and the adverse effects of UM through counselling in the antenatal period positively influenced pregnant women on safe drug use [51]. Additionally, grand multiparous women had an increased likelihood of UM use. This finding has been supported by other studies [3, 48, 54], and it is thought that these women's drug use experience in previous pregnancies makes them less cautious about drugs compared to earlier pregnancies. This suggests the need for continuous health education, counselling and other behavioural change communication strategies like posters, health talks and radio messages to pregnant women, and offering family planning to grand multiparous women during ANC. Notably, antenatal hospital admission increased the risk of UM use by almost five-fold in our study population. Perhaps, these women had to result in UM use because of their severe ailments during pregnancy. Studies have shown that women with serious illnesses are likely to use UM because of the issues of cost and influence from others [3]. Suggesting the need for continuous health information on drug use during pregnancy. However, after adjusting for confounding variables, only higher education (AOR = 0.23) and the use of at least two doses of IPT-SP remained significantly associated with UM use.

Similarly, the prevalence of herbal medicine use was 21.7%, which is considerably lower than the prevalence reported by other studies in Nigeria [20, 55, 56]. Differences in prevalence may be due to study setting and time, methodology and measurement issues and study characteristics. A high prevalence of HM during pregnancy is hazardous to the mother and infant because of the potential side effects, safety concerns and the lack of safety data. Also, women wrongly perceive HM as safe because they are natural products [19, 55]. The lack of regulation of HM use and unrestricted sale and advertisement in Nigeria make HM accessible and widely available in Nigeria [20, 55]. Meanwhile, health care providers pay little attention to HM use in pregnancy during ANC. In this study, women who used HM during pregnancy were generally older, less educated, unmarried, low income, had higher parity and had fewer ANC visits. The most familiar HM used among our study participants were herbal concoctions (which contained leaves, tree bark, and some unknown components), which has also been reported in Ghana [57]. Our study differs from other studies that reported bitter leaf, ginger, and garlic [20, 39, 56] as the commonest HM by pregnant women. The safety concerns of HM are related to its active ingredients, interaction with other drugs, the presence of contaminants, and lack of clinical trials to test its efficacy and safety [19, 56].

The factors associated with HM use in this study were similar to those of UM. On bivariate analysis, religion, income and number of ANC visits, IPT-SP use and antenatal hospital admission were associated with maternal HM use. Studies from Uganda [18] and Ethiopia [39] also reported similar factors, including low education, low monthly household income, rural dwellings etc. Specifically, women with low income, i.e. earning less than 20,000 Naira per month (the Nigerian minimum wage), were more likely to use HM during pregnancy as a result of high health care, lack of access to a health insurance scheme, and the lower cost of HM compared to orthodox medicines. These findings have been reported in the literature [18, 56]. We also found that women who attended at least four antenatal visits and used at least two doses of IPT-SP were less likely to use HM. Hence, ANC should provide the platform for health workers to engage pregnant women on their use of HM and provide evidence-based information on the use and adverse effects of herbal products and medicines. Additionally, hospitalised women during the antenatal period had a six-fold risk of HM use, suggesting that women with severe conditions during pregnancy may likely complement their treatment with HM [19]. On multivariate analysis, all these factors, however, became statistically significant. However, a lack of statistical significance does not diminish their clinical or public health relevance.

Generally, the pregnancy outcomes associated with unprescribed medicines and HM have been sparsely investigated, particularly in LMIC. The prospective cohort study design in this study facilitated the investigation of the pregnancy outcomes associated with UM and HM. Generally, we observed that women who used UM or HM had higher incidences of macrosomia, birth asphyxia and postpartum haemorrhage among our study participants. However, on bivariate analysis, the relative risk of caesarean section was 39% lower among women who had UM than women without UM. The explanation for this finding may not be as clear. This association, however, became insignificant after adjusting for background factors. Some other studies have found no increased risk of adverse pregnancy outcomes among pregnant women using certain medications [16, 49, 58]. A possible explanation may be that the unprescribed drugs used by women in our population were generally safe and did not contain high-risk medications. Unexpectedly, there were no adverse pregnancy outcomes reported among Turkish women who had inadvertently used drugs that are contraindicated in pregnancy (category X) [59]. Nonetheless, some studies (with substantial sample sizes) in Scotland [42], Sweden [27] and India [60] were able to demonstrate a positive association between UM and adverse pregnancy outcomes. Our study did not find any significant relationship between HM and pregnancy outcome. Similarly, some studies have reported no association between HM (or complementary medicines) and adverse pregnancy outcomes. A systematic analysis of complementary and alternative medicine use among pregnant women found no increased risk of adverse birth events among women who used herbs or natural medicine [61].

Our study's main strength is exploring the pregnancy outcomes associated with UM and HM in Nigeria; previous studies primarily examined the prevalence and associated factors. The prospective cohort study design minimised temporality bias. While the use of multiple health facilities also enhanced the generalizability of the study. Our study also examined the influence of substances like alcohol and tobacco exposure, providing empirical evidence for promoting a healthy lifestyle among pregnant women. However, our study still has some limitations. First, the response rate for maternal drug utilization was low compared to the initial cohort. Despite this, the study still had sufficient power to examine the associations. The self-reported assessment of UM and HM may introduce measurement error from under-reporting and a social desirability bias. Also, the external generalizability may not involve pregnant women in rural areas. Our study was also not sufficiently powered to examine the association of specific medications or herbal remedies with pregnancy outcomes. Therefore, we suggest that future research should conduct studies with sufficient sample sizes to examine the associations of specific medications with pregnancy outcomes.

## Conclusions

The prevalence of unprescribed medicine use and herbal medicine use was high among pregnant women in Nigeria. Analgesics, supplements and anti-malarial medicines were the most frequently used unprescribed medicines among pregnant women. Conversely, herbal remedies with uncertain safety profile was most commonly used in our study population. We observed that pregnant women who had high level of education and used more than 2 doses of IpTP-SP were less likely to use unprescribed medicines, while women who earned above 20, 000 monthly, had more than 4 ANC visits, and more than 2 doses of IpTP-SP were less likely to use herbal medicines. Use of unprescribed medicines and herbal medicines were not significantly associated with adverse pregnancy outcomes. The significance of medication use during pregnancy particularly unprescribed and herbal medications, should be emphasized Nigeria’s maternal health services particularly during antenatal care.

## Data Availability

The Ibadan Pregnancy Cohort Study datasets generated and analysed during the current study are not publicly available because they contain potentially identifying and confidential information but are available from the UI/UCH Ethics Committee (uiuchec@gmail.com) on reasonable request if it meets the criteria for accessing confidential data.
